# Azilsartan as “Add-On” Treatment with Methotrexate Improves the Disease Activity of Rheumatoid Arthritis

**DOI:** 10.1155/2018/7164291

**Published:** 2018-05-15

**Authors:** Naza Mohammed Ali Mahmood, Saad Abdulrahman Hussain, Hawar Ali Ehsan Kaka Khan

**Affiliations:** ^1^Department of Pharmacology and Toxicology, College of Pharmacy, University of Sulaimani, Kurdistan Region Sulaimani, Iraq; ^2^Department of Pharmacology and Toxicology, Faculty of Pharmacy, Al-Rafidain University College, Baghdad, Iraq; ^3^Specialized Center of Rheumatology, Kurdistan Region, Sulaimani, Iraq

## Abstract

**Objective:**

The present study aimed to evaluate the efficacy and safety of azilsartan (Azil) as “add-on” treatment with methotrexate (MTX) in patients with active rheumatoid arthritis (RA).

**Methods:**

This single center, randomized, placebo-controlled, double-blind, pilot study included 64 patients with active RA. Patients received either placebo or Azil in addition to their currently used MTX doses for 90 days. The primary outcomes were DAS-28, SDAI, HAQ-DI, CDAI, EGA, and swollen and tender joints count. The secondary outcomes were the changes in the pain visual analogue scale (VAS-100), serum levels of TNF-*α*, IL-1*β*, IL-6, and anti-CCP, the lipid profile, and the markers of kidney and liver functions in the two groups at baseline and after 90 days.

**Results:**

After 90 days, most clinical scores were significantly better in the Azil-treated group than in the placebo group. All inflammatory biomarkers were significantly improved after treatment with MTX + Azil compared to baseline and placebo group. No safety concerns were reported during the study period.

**Conclusions:**

Azilsartan improved the effects of methotrexate on the clinical scores and certain inflammatory biomarkers of patients with active RA.

**Trial Registration:**

The protocol was registered under the number 507/SA/1024 at the local clinical studies database, College of Medicine, Sulaimani University.

## 1. Introduction

Rheumatoid arthritis (RA) is a chronic, systemic pathological disorder described as persistent inflammation of the synovial joints. Uncontrolled active RA leads to severe joint damage, which may progress to disability, poor quality of life, and other comorbid conditions such as cardiovascular diseases [1]. Although steroids and nonsteroidal anti-inflammatory drugs (NSAIDs) are recommended for the treatment of joint pain and other symptoms of systemic inflammation associated with RA, chronic use of these drugs was not considered as a current practice and was associated with serious adverse effects that were poorly tolerated by most of RA patients [2]. Meanwhile, many therapeutic approaches are currently used in clinical practice to treat active RA, including disease-modifying drugs (DMARDs), like methotrexate (MTX) and biological agents; however, rapport between their long-term use and wide range of side effects, in addition to high cost, limits their use, especially in low-income communities [3, 4]. Added to the central role of the immune system in the inflammatory response, increased expression of type-1 angiotensin II receptors (AT1-R) was found to be involved in various chronic inflammatory disorders [5]. Moreover, stimulation of AT1-R was associated with excessive production of reactive oxygen species (ROS) and enhanced secretion of inflammatory cytokines that accelerate inflammatory cascades [6, 7]. These findings are supported by the fact that AT1-R blockade attenuated inflammatory response in animal models of inflammatory liver diseases [8, 9]. Furthermore, excessive activation of the renin-angiotensin system (RAS) mediates inflammation and modulates the immune response of T-cells, suggesting a potential influence in autoimmune diseases such as RA [10, 11]. In this regard, several studies revealed the beneficial role of angiotensin receptor blockers (ARBs) and angiotensin-converting enzyme inhibitors (ACEIs) in experimental animal models of arthritis [12, 13]. It is noteworthy that newly approved ARBs, such as telmisartan and azilsartan, demonstrated potent anti-inflammatory activity in animal models through mechanisms not related to RAS blockade [14, 15]. Yet, there are no data available from randomized clinical trials to support this concept. In light of this indirect evidence, blockade of AT1-R results in dual antihypertensive and anti-inflammatory effects; this may be an effective therapeutic choice. Even though the use of ARBs is not expected to replace antirheumatic drugs such as MTX and biological agents, they may be suggested as an adjunct therapy to improve response in RA patients. Accordingly, we design this pilot clinical study to assess, for the first time, the clinical beneficial effects of the ARB azilsartan as an adjuvant treatment with MTX in patients with active RA.

## 2. Materials and Methods

### 2.1. Patient Recruitment and Study Design

**Figure 1:**
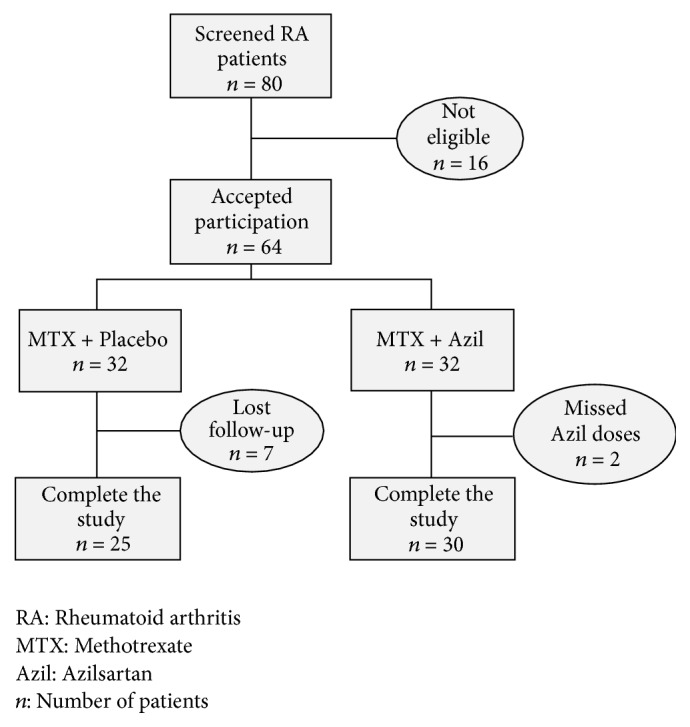
Flowchart of the study.

We performed a double-blinded, placebo-controlled, randomized pilot clinical study with treatment duration of 90 days over 14 months (from April 2016 to June 2017) at the Specialized Center of Rheumatology, Sulaimani City, Kurdistan Region, Iraq. Eighty patients with active RA, who routinely went to the Specialized Rheumatology Center for treatment follow up, were screened for eligibility. Based on the 2010 ACR/EULAR criteria [16] and 28-joint Disease Activity Score (DAS-28) ≥ 3.2, only 64 patients with active moderate to severe RA were enrolled in the study (age: range 20–70 years). All the enrolled patients demonstrate poor response to the currently used oral methotrexate (MTX) (doses ranged between 7.5 mg/week and 25 mg/week for at least 3 months) at the time of screening eligibility for inclusion and were candidate for initiation of treatment with biologic DMARDs as “add-on” approach. Only 55 patients completed the study: the evaluators lost the contact with 7 patients in the MTX-placebo group during week 3 and week 5 for unknown reasons, while 2 patients in the MTX-Azil group were excluded due to missing more than 2 doses of azilsartan (Figure 1). In a double-blinded pattern, the patients were randomly assigned to either of two treatment groups in an approximate 2 : 2 ratio: Methotrexate plus placebo treated (MTX + placebo; *n* = 32) or to methotrexate plus Azilsartan-treated (MTX + Azil; *n* = 32). Methotrexate (Ebewe Pharma, Austria) was already administered as an oral tablet (7.5–25 mg per week) as a part of their treatment program before inclusion, while azilsartan was administered as a single oral daily dose (20 mg/day). The azilsartan (Apollo Healthcare Resources, Singapore) doses were prepared as a capsule dosage form and administered as “add-on” single daily doses with the regularly used MTX regimen. The placebo dose was prepared as a capsule dosage form that matches the shape and color of the test drug formula and administered similarly. The patients were advised to keep on their regular drug treatment schedule and were regularly observed clinically every 15 days for proper compliance and occurrence of any unusual adverse effects. Before inclusion, all randomized patients were asked to sign informed consent form according to the principles of the Declaration of Helsinki. The local scientific ethics committee of the University of Sulaimani, College of Medicine approved the study protocol (Certificate number 507/SA/1024).

### 2.2. Outcome Measurement and Follow Up

At the time of inclusion, patients with one of the following characteristics were excluded: patients with mild or inactive RA, patients using nonsteroidal anti-inflammatory drugs 2 days before inclusion, hypersensitivity or severe adverse effects to the tested drugs, impaired renal or hepatic function, pregnant and breastfeeding women, juvenile RA, patients using disease-modifying anti-rheumatic drugs other than MTX, biologics or high-dose steroids (>10 mg/day prednisolone or equivalent), hypertensive patients using ACEIs, ARBs or any drug that interfere with RAS for treatment of hypertension, and coexistence of other connective tissue diseases. For assessment of the clinical endpoints at baseline and at the end of 90-day treatment, 4 instruments of clinical outcome evaluation were used including the Disease Activity Score-28 joint (DAS-28) [17], simplified disease activity index (SDAI) [18], clinical disease activity index (CDAI), and the health assessment questionnaire disease index (HAQ-DI) that assesses functional ability for eight subscales: arising, common daily activities, dressing, eating, grip, hygiene, reach, and walking [19]. Additionally, tender joints count (TJC), swollen joints count (SJC), pain severity using visual analogue scale (VAS-100), evaluator global assessment (EGA), and duration of morning stiffness (measured in minutes) were also evaluated to support the clinical assessment primary outcomes. Blood samples (10 ml) were obtained from each patient by vein puncture at baseline and the end of the treatment period. Of the blood collected, 3 ml was kept in an ethylene diamine tetra-acetic acid tube to be used for measurement of erythrocyte sedimentation rate (ESR) and hematology markers. The remaining blood was kept in a plain tube, left to clot at room temperature for 30 min, and centrifuged for 10 min at 4000 rpm to get the serum. Using ready-made enzyme-linked immunosorbent assay kits, the resultant serum was utilized for the measurement of highly sensitive C-reactive protein (hs-CRP) (Demeditec, Germany), tumor necrosis factor-*α* (TNF-*α*), interleukins-1*β* and -6 (IL-1*β*, IL-6), and anticyclic citrullinated peptide (anti-CCP) (Beckman Coulter, USA). Additionally, the lipid profile (Triglycerides: TG, total cholesterol: TC, low-density lipoprotein cholesterol: LDL-c and high-density lipoprotein cholesterol: HDL-c) and the markers of hepatic and renal functions (urea, creatinine, aspartate aminotransferase: AST and alanine aminotransferase: ALT) were analyzed spectrophotometrically using ready-made kits (Biomerieux, France).

### 2.3. Statistical Analysis

The results were statistically analyzed utilizing Graph Pad Prism 5.1 software (Graph Pad Software Inc., California, US). Continuous variables were presented as mean ± S.D, while discrete variables presented as numbers and frequencies. The Chi-square and Wilcoxon-rank tests were used to test the significance of the association between discrete variables. Paired *t*-test was used to evaluate the difference between pre- and posttreatment values. Additionally, one-way ANOVA was used to evaluate the significance of the difference between means and supported by Bonferroni's post hoc analysis. Values with *P* < 0.05 were considered significantly different.

## 3. Results

### 3.1. Primary Outcome: Clinical Scores

Before initiation of the treatment, the demographic characteristics of the patients were recorded; the results revealed no significant differences between the two randomized patients groups as shown in Table 1. Although no significant difference was reported for DAS-28 score at baseline, our data showed that adjunct use of Azil with MTX significantly decreased the DAS-28 score after 90 days compared with baseline value (5.2 ± 0.8 versus 6.5 ± 0.7; *P* < 0.001), and this effect was found to be significantly greater than that of the placebo (Table 2). Similarly, the SDAI score was not significantly decreased in the MTX + placebo treated group compared with baseline, while coadministration of Azil with MTX decreases significantly the SDAI score compared with both the baseline and the placebo group (43.3 ± 15.8 versus 62.8 ± 13.4 and 61.5.5 ± 19.6, resp.; *P* < 0.01) at the end of the treatment period. Regarding the effects on HAQ-DI score, Table 2 showed that adjunct use of Azil with MTX decreases significantly the HAQ-DI score after 90 days of treatment (1.3 ± 0.6 versus 1.8 ± 0.5; *P* = 0.001), and this value was significantly greater than that of the placebo group as well. However, the addition of the placebo did not significantly change this score compared with baseline. Moreover, Table 2 showed that treatment with Azil decreases significantly the CDAI score (37.5 ± 15.8 versus 56.3 ± 11.2; *P* < 0.001) after 90 days of treatment, and this effect was significantly greater than that reported in the placebo group. Table 2 also showed that Azil significantly improved the TJC score compared with the baseline value (18.1 ± 6.7 versus 25.3 ± 3.1; *P* < 0.01); however, the posttreatment value was not significantly different compared to the placebo group. Meanwhile, combination of MTX with Azil decreased SJC significantly after 90 days compared with baseline (11.4 ± 8.8 versus 17.3 ± 9.7; *P* = 0.0004) and placebo group, whereas coadministration of the placebo did not significantly alter this score compared to baseline. The influence of Ang II receptor blockade on the VAS-100 score was also examined, where coadministration of Azil with MTX significantly improved the VAS-100 score compared to baseline value (49.3 ± 17.5 versus 70.0 ± 13.6; *P* < 0.001); this pattern of effect was not recognized in the placebo treated group (*P* > 0.05) after 90 days. Table 2 also showed that the EGA score in Azil-treated group was significantly decreased compared to both baseline value and placebo group after 90 days (4.1 ± 1.1 versus 6.2 ± 1.1 and 6.8 ± 1.1, resp.; *P* < 0.001), while coadministration of the placebo formula with MTX did not significantly influence the EGA score at the end of the treatment period. Moreover, the MTX-Azil combination improved the duration of morning stiffness significantly compared to both the baseline value and the MTX-placebo effect after 90 days (16.6 ± 5.9 versus 26.1 ± 8.5 and 19.2 ± 4.9, resp.; *P* = 0.001), where the placebo formula did not show significant effect in this regard (Table 2). The ESR value in MTX-Azil group was significantly decreased after 90 days compared to baseline (28.5 ± 11.0 versus 37.6 ± 15.8; *P* = 0.0006), while coadministration of the placebo with MTX did not show such effect, and the ESR value was significantly greater than that of the MTX-Azil group posttreatment (*P* = 0.72) as shown in Table 2. Furthermore, serum hs-CRP levels did not significantly change (*P* = 0.62) in both treatment approaches compared to baseline values. The influence of coadministration of either Azil or placebo with MTX on different functional areas of the HAQ-DI score was demonstrated in Table 3. Although all areas of the HAQ-DI score were significantly improved in the MTX-Azil-treated group compared to baseline (*P* < 0.001), only those that represent arise, eat, walk, and hygiene areas demonstrated significant differences compared to that reported in the MTX-placebo treated group posttreatment.

### 3.2. Secondary Outcome: Biomarkers of Inflammation

In Table 4, the results indicated that coadministration of Azil with MTX significantly reduced the serum concentration of TNF-*α* after 90 days compared to the baseline value (14.2 ± 4.2 versus 18.4 ± 5.5 pg/ml; *P* < 0.001), and this level was significantly lower than that reported in the placebo group posttreatment; however, no significant differences were reported for serum TNF-*α* levels between the two groups at baseline. Similarly, serum concentration of IL-1*β* was not significantly changed in the MTX + placebo treated group compared to the baseline, while coadministration of Azil with MTX significantly decreases serum IL-1*β* compared to both baseline and MTX + placebo treated group (11.6 ± 5.4 versus 16.1 ± 3.9 and 16.9 ± 4.4 pg/ml, resp.; *P* = 0.001) at the end of the treatment period. Table 4 also revealed that the serum concentrations of IL-6 in the MTX-placebo group were nonsignificantly changed compared to baseline values (*P* = 0.26), while coadministration of Azil with MTX resulted in significant decrease in serum IL-6 levels compared to both baseline values and MTX + placebo group posttreatment (16.7 ± 4.2 versus 23.9 ± 5.6 and 30.3 ± 7.1 pg/ml, resp.; *P* < 0.001). Serum anti-CCP concentrations were significantly decreased in the MTX + placebo treated group compared to baseline; likewise, coadministration of Azil with MTX resulted in significant decrease in serum anti-CCP concentrations compared to both baseline values and MTX + placebo treated group (69.9 ± 9.6 versus 88.6 ± 9.3 and 84.5 ± 8.3 *μ*g/ml, resp.; *P* = 0.001) at the end of the treatment period.

### 3.3. Lipid Profile

The results indicated that coadministration of Azil with MTX produced significant decrease in TG and LDL-c concentrations, associated with significant increase in HDL-c levels compared to baseline values; however, these changes were not significantly different compared to the MTX + placebo group posttreatment (Table 5).

### 3.4. Safety Profile

The influence of using Azil with MTX on the hepatic and renal functions in RA patients was shown in Table 6. Serum levels of AST and ALT were significantly elevated in the MTX + placebo group compared to baseline, while adjunct use of Azil with MTX resulted in significant decrease in the AST levels compared to baseline. However, serum urea and creatinine levels were not significantly changed in the two groups. Regarding the hematopoietic system, coadministration of Azil with MTX did not significantly change Hb levels, while WBC count was significantly decreased (*P* < 0.05) in the MTX + Azil group compared to baseline; however, it was comparable to MTX + placebo group (Table 7). Although both groups demonstrated significant decrease in the platelets count posttreatment, these changes were comparable (*P* > 0.05).

## 4. Discussion

Increasing evidence emerged regarding the role of RAS activation in inflammatory disorders, where upregulation of Ang II type-1 receptors in the synovium of RA patients was found to play a role in this context [20]. The beneficial effect of Azil on some inflammatory disorders was previously demonstrated, as both prophylactic and therapeutic administration significantly decreased inflammatory consequences in animal models of inflammation [15]. However, the coadministration of MTX and Azil was not evaluated clinically or in animal models of RA. Apart from the cardiovascular modulatory effects, certain ARBs, like losartan and telmisartan, have well-characterized anti-inflammatory activities [21, 22]. Based on data that suggested the anti-inflammatory activity of azilsartan [15], we assessed the influence of adjunct use of azilsartan with MTX on the clinical and biochemical markers in RA patients. The results of this pilot clinical study demonstrated, for the first time, that use of Azil as “add-on” option with MTX in the treatment of patients with active RA augments the effects of the latter in improving the biochemical and clinical progression of the disease. Our results regarding the beneficial effects of Azil, as adjunct treatment with MTX, in improving the clinical scores and the inflammatory cytokines levels in RA patients were in tune with the previously reported data about the anti-inflammatory properties of several RAS modulators* in vitro* and* in vivo* [23, 24]. We have recently reported the anti-inflammatory effect of Azil, when used alone or in combination with the direct renin inhibitor (aliskiren), to attenuate the production of TNF-*α* and IL-1*β* in a rat model of high fat diet-induced steatohepatitis [25]. Based on our previous results and others that evaluated coadministration of losartan with MTX in animal model of RA [25, 26], we accelerated our efforts toward conducting the present pilot clinical study. Our study revealed that Azil may improve the inhibitory effects of MTX on tissue destruction attributed to the excessive production of damaging inflammatory mediators within the inflamed joint tissues. The results of the present study are consistent with previously reported data, where Azil or other ARBs decreased synthesis of the proinflammatory cytokines by various mechanisms including Ang II type-1 receptor blockade and/or activation of PPAR-*γ* nuclear receptors [27]. In our study, Azil enhanced the antirheumatic effect of MTX, manifested as a significantly greater improvement in the clinical and biochemical markers compared to the use of MTX with the placebo formula. Although the benefit of using Azil alone was not assessed due to ethical considerations, its concomitant use with other antiarthritic agents like biologic agents could show promising results (unpublished data), and future studies with larger patients sample and longer duration are proposed to elucidate its therapeutic role in this regard. In the present study, coadministration of Azil with MTX demonstrated quantitatively different outcomes in the disease activity indices. Although all scores were significantly improved compared to placebo, the level of changes at the end of the treatment was varied and significantly better than that reported in the MTX + placebo group. The differences in the result of these scores and the effect of Azil depend on multiple factors including the tender and swollen joint counts, pain severity, and the ESR value. The antiarthritic role of Azil in RA patients could be linked to the attenuation of cytokines production including TNF-*α*. This effect supports other previously reported data regarding the use of RAS modulators (ACEIs and ARBs) in inflammatory conditions [28, 29]. Moreover, Azil improves the ability of MTX to decrease the levels of inflammatory markers like TNF-*α* and anti-CCP antibodies. The detection of anti-CCP antibodies in the serum of RA patients depends on their immunoreactivity with many cyclic citrullinated peptide fragments of natural human proteins and correlated with the severity of RA and synovial tissue damage [30, 31]. It has been demonstrated that elevation of anti-CCP antibodies is significantly correlated with the excessive activation of phospholipase A2 and C-reactive protein at the preclinical period of RA [32]. In the present study, the reported improvement of anti-CCP levels in the Azil-treated group possibly attributed to the blockade of Ang II-mediated increase of phospholipase A2 activity during excessive RAS activation [33]. Consistent with previous studies, our results addressed the role of RAS blockade in RA treatment; however, concomitant use of Azil with MTX was not evaluated with regard to the efficacy and safety of the MTX in animal models or human studies [34, 35]. Coadministration of ARBs, like Azil, with MTX could be of value in limiting the adverse effects of MTX or other DMARDs drugs used for the RA treatment and may influence the increasing cost and inadequate patient compliance related to emergence of the adverse effects. The present study revealed that the extent of changes in the clinical scores was not associated with improving all the inflammatory biomarkers. This behavior is quite usual when using drugs that target TNF-*α* during RA treatment [36], and the relation between the biochemical and clinical outcomes of the treatment approach used in the present study was affected by the study limitations. The relatively moderate benefits of Azil reported in this pilot study, which involved Iraqi patients with active RA, could be due to its pleiotropic effects that interfere with multiple pathological events of RA including anti-inflammatory and immunomodulatory activities. Meanwhile, other studies reported the dose-dependent antioxidant and anti-inflammatory activities of Azil, and many of these activities were produced by using lower daily doses, compared to the dose used in our study [23, 37]. Moreover, the sample size limitation and short duration of treatment should be considered as important factors that variably influence clinical and biochemical outcomes of the present study. Larger sample and longer clinical trial duration are highly recommended.

## 5. Conclusion

For the first time, we reported that azilsartan may improve the effects of methotrexate on the clinical scores and certain inflammatory biomarkers of patients with active RA.

## Figures and Tables

**Table 1:** Demographic data and baseline characteristics of the RA patients treated with methotrexate (MTX) or its combination with azilsartan (Azil).

Parameters	MTX + placebo	MTX + Azil	*P* value
	*n* = 25	*n* = 30	
*Gender*			
Male *n* (%)	8 (32)	12 (40)	0.62
Female *n* (%)	17 (78)	18 (60)	0.53
Age (years)	55.5 ± 12.8	68.1 ± 13.0	0.57
Body weight (Kg)	82.7 ± 10.5	81.2 ± 9.6	0.41
BMI (Kg/m^**2**^)	31.9 ± 8.5	30.5 ± 6.9	0.62
Disease duration (year)	10.8 ± 9.1	8.7 ± 5.3	0.44
MTX treatment (months)	28.5 ± 12.8	25.4 ± 11.3	0.45
ESR (mm/hr)	36.6 ± 8.6	37.6 ± 15.8	0.84
hsCRP (*μ*g/ml)	6.5 ± 3.2	5.7 ± 3.9	0.55
DAS-28 score (4 values)	6.4 ± 0.96	6.5 ± 0.77	0.96
SDAI score	63.2 ± 20.1	62.8 ± 13.4	0.94
HAQ-DI score	1.77 ± 0.6	1.83 ± 0.58	0.7
CDAI score	51.6 ± 15.3	56.3 ± 11.2	0.33
Joint deformities *n* (%)	4 (16.0)	5 (16.7)	0.48
Use of steroids *n* (%)	3 (12)	4 (13.3)	0.5
Use of NSAIDs *n* (%)	2 (8)	2 (6.7)	0.61
*Associated diseases*			
Hypertension *n* (%)	4 (16.0)	6 (20.0)	0.12
Diabetes mellitus *n* (%)	3 (12.0)	2 (6.7)	0.11

Values are presented as mean ± S.D or percentage; *n*: number of patients; MTX: methotrexate; Azil: azilsartan; NSAIDs: nonsteroidal anti-inflammatory drugs.

**Table 2:** Effect of azilsartan (Azil) and placebo on ESR and hs-CRP levels and the clinical evaluation scores of patients with active RA maintained on methotrexate (MTX).

Clinical Score	MTX + placebo, *n* = 25		MTX + Azil, *n* = 30	
	*Baseline*	*After 90 days*	*Baseline*	*After 90 days*
DAS-28	6.44 ± 0.9^a^	6.49 ± 0.8^a^	6.45 ± 0.7^a^	5.2 ± 0.8^*∗*b^
SDAI	63.2 ± 20.1^a^	61.5 ± 19.6^a^	62.8 ± 13.4^a^	43.3 ± 15.8^*∗*b^
HAQ-DI	1.8 ± 0.6^a^	2.0 ± 0.6^*∗*a^	1.8 ± 0.5^a^	1.3 ± 0.6^*∗*b^
CDAI	51.6 ± 15.3^a^	50.0 ± 15.3^a^	56.3 ± 11.2^a^	37.5 ± 15.8^*∗*b^
TJC-28	23.2 ± 7.6	22.9 ± 7.5	25.8 ± 3.1	18.1 ± 6.7^*∗*^
SJC-28	16.0 ± 9.3^a^	15.8 ± 9.4^a^	17.3 ± 9.7^a^	11.4 ± 8.8^*∗*b^
Pain VAS-100 (mm)	71.9 ± 12.2^a^	69.8 ± 12.9^a^	70.0 ± 13.6^a^	49.3 ± 17.5^*∗*b^
EGA (cm)	6.7 ± 1.3^a^	6.8 ± 1.1^a^	6.2 ± 1.1^a^	4.1 ± 1.5^*∗*b^
Morning stiffness (min)	21.5 ± 11.6^a^	19.2 ± 4.9^a^	26.1 ± 8.5^a^	16.6 ± 5.9^*∗*b^
ESR (mm/hr)	36.6 ± 8.6^a^	37.2 ± 8.7^a^	37.6 ± 15.8^a^	28.5 ± 11.0^*∗*b^
hsCRP (*μ*g/ml)	6.5 ± 3.3	6.02 ± 3.6	5.7 ± 3.9	4.9 ± 2.8

Values are presented as mean ± SD; *n* = number of patients; ^*∗*^significantly different compared to pretreatment within the same group (*P* < 0.050); values with different superscripts (a, b) among groups are significantly different (*P* < 0.05). RA: rheumatoid arthritis; TJC: tender joint count; SJC: swollen joint count; VAS: visual analogue scale; EGA: evaluator global assessment; ESR: erythrocyte sedimentation rate; hsCRP: highly sensitive C-reactive protein; DAS-28: 28-joint disease activity score; SDAI: simple disease activity index; HAQ-DI: health assessment questionnaire disability index; CDAI: clinical disease activity index.

**Table 3:** Effect of azilsartan (Azil) on different functional areas of HAQDI score of patients with active RA maintained on methotrexate (MTX).

HAQDI Areas	MTX + placebo (*n* = 25)		MTX + Azil (*n* = 30)	
	*Baseline*	*After 90 days*	*Baseline*	*After 90 days*
Dress	1.6 ± 1.1	1.7 ± 1.0	1.4 ± 0.9	0.8 ± 0.8^*∗*^
Arise	2.0 ± 0.5^a^	2.2 ± 0.4^*∗*a^	1.7 ± 0.9^a^	1.1 ± 0.7^*∗*b^
Eat	2.0 ± 0.5^a^	2.3 ± 0.6^*∗*a^	2.3 ± 0.9^a^	1.5 ± 0.9^*∗*b^
Walk	2.2 ± 0.7^a^	2.5 ± 0.5^*∗*a^	2.1 ± 0.8^a^	1.4 ± 0.9^*∗*b^
Hygiene	1.6 ± 0.8^a^	1.7 ± 0.7^a^	1.3 ± 0.7^a^	1.0 ± 0.6^*∗*b^
Reach	2.1 ± 0.6	2.2 ± 0.7	2.3 ± 0.8	1.8 ± 0.8^*∗*^
Grip	1.4 ± 0.5	1.7 ± 0.8^*∗*^	1.1 ± 1.9	0.7 ± 1.5^*∗*^
Daily activity	1.9 ± 0.8	2.0 ± 0.8	2.1 ± 0.9	1.5 ± 0.9^*∗*^

Values are expressed as mean ± S.D; *n*: number of patients; ^*∗*^significantly different compared to pretreatment (*P* < 0.05); posttreatment values with different superscripts (a, b) within each parameter are significantly different (*P* < 0.05). HAQ-DI: health assessment questionnaire disability index; RA: rheumatoid arthritis.

**Table 4:** Effect of azilsartan (Azil) and placebo on TNF-*α*, IL-1*β*, IL-6 and anti-CCP levels and the clinical evaluation scores of patients with active RA maintained on methotrexate (MTX).

Inflammatory marker	MTX + placebo, *n* = 25		MTX + Azil, *n* = 30	
	*Baseline*	*After 90 days*	*Baseline*	*After 90 days*
TNF-*α* (pg/ml)	17.8 ± 5.1^a^	16.7 ± 5.6^a^	18.4 ± 5.5^a^	14.2 ± 4.2^*∗*b^
IL-1*β* (pg/ml)	15.3 ± 2.9^a^	16.9 ± 4.4^a^	16.1 ± 3.9^a^	11.6 ± 5.4^*∗*b^
IL-6 (pg/ml)	29.1 ± 5.3^a^	30.3 ± 7.1^a^	23.9 ± 5.6^b^	16.7 ± 4.2^*∗*c^
Anti-CCP (IU/ml)	90.4 ± 10.0^a^	84.5 ± 8.3^*∗*b^	88.6 ± 9.3^a^	69.9 ± 9.6^*∗*c^

Values are presented as mean ± SD; *n* = number of patients; ^*∗*^significantly different compared to pretreatment within the same group (*P* < 0.050); values with different superscripts (a, b, and c) among groups are significantly different (*P* < 0.05).

**Table 5:** Effect of azilsartan (Azil) on serum lipid profile of patients with active RA maintained on methotrexate (MTX).

Parameters	MTX + placebo (*n* = 25)		MTX + Azil (*n* = 30)	
	*Baseline*	*After 90 days*	*Baseline*	*After 90 days*
Triglycerides (mg/dl)	143.3 ± 31.7	147.6 ± 36.6	153.2 ± 32.9	122.0 ± 34.4^*∗*^
Cholesterol (mg/dl)	170.5 ± 32.0^a^	181.9 ± 25.5^a^	165.1 ± 23.8^a^	154.3 ± 29.4^a^
LDL-c (mg/dl)	100.9 ± 29.3	105.8 ± 24.7	105.4 ± 19.2	94.4 ± 15.8^*∗*^
HDL-c (mg/dl)	39.4 ± 9.6	38.7 ± 7.9	33.3 ± 9.4	38.0 ± 11.9^*∗*^

Values are presented as mean ± S.D; *n*: number of patients; ^*∗*^significantly different compared with baseline within the same group (*P* < 0.05); values with different superscripts (a, b) within each parameter are significantly different (*P* < 0.05). LDL-c: low-density lipoprotein cholesterol; HDL-c: high-density lipoprotein cholesterol.

**Table 6:** Effect of azilsartan (Azil) on the liver and kidney function markers of patients with active RA maintained on methotrexate (MTX).

Parameters	MTX + placebo (*n* = 25)		MTX + Azil (*n* = 30)	
	*Baseline*	*After 90 days*	*Baseline*	*After 90 days*
Serum AST (U/L)	19.9 ± 3.8^a^	30.6 ± 7.2^*∗*b^	19.8 ± 3.7^a^	17.6 ± 3.2^*∗*c^
Serum ALT (U/L)	19.7 ± 7.4^a^	30.3 ± 12.1^*∗*b^	17.0 ± 4.6^a^	15.3 ± 5.4^a^
Serum creatinine (mg/dl)	0.63 ± 0.1^a^	0.67 ± 0.1^a^	0.61 ± 0.2^a^	0.54 ± 0.1^a^
Serum urea (mg/dl)	27.4 ± 7.7^a^	29.6 ± 7.2^a^	23.9 ± 9.3^a^	21.3 ± 6.8^a^

Values were presented as mean ± S.D; *n*: number of patients; ^*∗*^significantly different compared with baseline within the same group (*P* < 0.05); values with different superscripts (a, b, and c) within each parameter were significantly different (*P* < 0.05).

**Table 7:** Effect of azilsartan (Azil) on the hematopoietic function markers of patients with active RA maintained on methotrexate (MTX).

Parameters	MTX + placebo (*n* = 25)		MTX + Azil (*n* = 30)	
	*Baseline*	*After 90 days*	*Baseline*	*After 90 days*
Hb (g/dL)	12.4 ± 1.1^a^	12.1 ± 1.2^a^	12.4 ± 1.2^a^	12.3 ± 1.1^a^
WBC count ×10^3^ cells/*μ*L	7.1 ± 1.4	6.7 ± 1.3	7.0 ± 1.8	6.2 ± 1.5^*∗*^
Platelets count ×10^9^ cells/L	272.2 ± 41.2	265.8 ± 43.6^*∗*^	265.6 ± 51.1	233.3 ± 40.2^*∗*^

Values were presented as mean ± S.D; *n*: number of patients; ^*∗*^significantly different compared with baseline within the same group (*P* < 0.05); values with different superscripts (a, b) within each parameter were significantly different (*P* < 0.05).

## Data Availability

The data used to support the findings of this study are available from the corresponding author upon request.

## Abbreviations

ACR: American College of Rheumatology
Ang II: Angiotensin II
Anti-CCP: Anti-cyclic citrullinated peptide
AT1-R: Angiotensin type-1 receptor
Azil: Azilsartan
CDAI: Clinical disease activity index
DAS-28: Disease Activity Score-28 Joints
DMARDs: Disease-modifying antirheumatic drugs
EGA: Evaluator global assessment
EULAR: European League Against Rheumatism
HAQ-DI: Health assessment questionnaire disease index
IL-1*β*: Interleukin-1-beta
IL-6: Interleukin-6
MTX: Methotrexate
RA: Rheumatoid arthritis
RAS: Renin-angiotensin system
ROS: Reactive oxygen species
SDAI: Simplified disease activity index
TNF-*α*: Tumor necrosis factor-alpha
VAS: Visual analogue scale.
